# A Cybersecurity NER Method Based on Hard and Easy Labeled Training Data Discrimination

**DOI:** 10.3390/s25247627

**Published:** 2025-12-16

**Authors:** Lin Ye, Yue Wu, Hongli Zhang, Mengmeng Ge

**Affiliations:** 1 School of Cyberspace Science, Harbin Institute of Technology, 150001 Harbin, China; 21b903103@stu.hit.edu.cn (Y.W.); zhanghongli@hit.edu.cn (H.Z.); 2 College of Computing and Data Science, Nanyang Technological University, 639798 Singapore, Singapore; mengmeng.ge@ntu.edu.sg

**Keywords:** deep learning, pre-trained language models, cybersecurity NER, hard and easy labeled training data, data augmentation

## Abstract

Although general-domain Named Entity Recognition (NER) has achieved substantial progress in the past decade, its application to cybersecurity NER is hindered by the lack of publicly available annotated datasets, primarily because of the sensitive and privacy-related nature of security data. Prior research has largely sought to improve performance by expanding annotation volumes, while overlooking the intrinsic characteristics of training data. In this study, we propose a cybersecurity Named Entity Recognition (NER) method based on hard and easy labeled training data discrimination. Firstly, a hybrid strategy that integrates a deep learning (DL)-based discriminator and a rule-based discriminator is employed to partition the original dataset into hard and easy samples. Secondly, the proportion of hard and easy data in the training set is adjusted to determine the optimal balance. Finally, a data augmentation algorithm is applied to the partitioned dataset to further improve model performance. The results demonstrate that, under a fixed total training data size, the ratio of hard to easy samples has a significant impact on NER performance, with the optimal strategy achieved at a 1:1 proportion. Moreover, the proposed method further improves the overall performance of cybersecurity NER.

## 1. Introduction

With the rapid development of the internet and the increasing sophistication of digital technologies, cybersecurity incidents have become a pervasive and pressing challenge. The frequency, diversity, and severity of cyberattacks continue to rise, leading to significant risks for governments, enterprises, and individuals alike. This trend is particularly evident in sensor-based systems and sensor networks, such as Internet of Things (IoT) deployments, industrial monitoring infrastructures, and intrusion detection sensors, which are widely deployed in modern cyber-physical environments.

These incidents not only reveal the evolving complexity of cyber threats but also result in the generation of an overwhelming volume of cybersecurity-related textual data [1]. Such data are embedded in diverse sources, ranging from threat intelligence reports and vulnerability disclosures to malware analyses and incident response documents, as well as logs, alerts, and event descriptions produced by sensor-based monitoring systems and sensor networks. Effectively mining these unstructured texts to extract meaningful and actionable information is crucial for improving situational awareness and enabling timely defense strategies. Among various natural language processing (NLP) tasks, NER plays a particularly vital role, as it supports the automatic extraction of entities such as malware names, IP addresses, software vulnerabilities, and threat actor groups, thereby facilitating structured knowledge construction in cybersecurity. In the context of sensor systems and sensor networks, these entities can further describe compromised sensor nodes, affected industrial devices, vulnerable services, and anomalies detected by intrusion detection sensors, which directly support attack detection and cyber defense in large-scale sensor deployments.

The advent of pre-trained language models (PLMs), including BERT [2], XLNet [3], RoBERTa [4,5], and GPT [6], has transformed the landscape of NLP by enabling the transfer of general-domain linguistic knowledge to specialized downstream tasks. In general-domain NER, these models are typically adapted through the pre-training and fine-tuning paradigm, in which a model is first trained on large-scale general-domain corpora and then fine-tuned on smaller, domain-specific datasets. This approach has consistently demonstrated strong performance across a wide array of NLP applications. However, its effectiveness in cybersecurity remains constrained by the unique challenges of this domain. Cybersecurity entities differ markedly from conventional ones such as names, locations, and dates: they are highly technical, context-sensitive, and rapidly evolving, often requiring precise interpretation within dynamic threat landscapes. This complexity introduces ambiguity and polymorphism, making entity recognition far more challenging than in general domains.

Several studies [7,8,9,10] have attempted to address these challenges. Alam et al. [11] introduced CyNER, an open-source library that integrates transformer-based models with existing NER systems to extract cybersecurity-specific entities from Cyber Threat Intelligence (CTI). While this work highlights the feasibility of applying PLMs in cybersecurity, it primarily focuses on fine-tuning large-scale models, overlooking the potential benefits of smaller and more efficient alternatives. Meanwhile, the NLP community has witnessed a growing interest in lightweight model variants designed to balance accuracy and efficiency. ALBERT [12], for instance, employs parameter sharing and factorized embeddings to reduce memory consumption, while TinyBERT [13] and MobileBERT [14] leverage knowledge distillation to compress large models without significant performance degradation. MiniLM [15] further combines structural optimization and distillation to achieve compact yet powerful models. These developments reflect a broader trend toward building models that can be deployed in real-world, resource-constrained environments, which is highly relevant for cybersecurity applications where computational efficiency is often essential. especially for sensor-based devices and edge nodes. Although distributed learning [16] and anonymized data protection [17] have been extensively studied in general domains, these approaches do not fully address the intrinsic sensitivity of cybersecurity information. From a fundamental perspective, deploying compact and privately maintained models provides a more reliable safeguard, as it minimizes the exposure of sensitive data and reduces reliance on external infrastructures.

Beyond model architecture, the quality and composition of training data represent another critical yet underexplored dimension. In vulnerability prediction research, prior work has examined the role of hard and easy negatives in model performance [18], demonstrating that training data difficulty can meaningfully affect outcomes. However, these studies typically rely on code snippets, where hard negatives are relatively easy to define (e.g., vulnerable code segments versus patched code). In contrast, cybersecurity NER operates on natural language texts, where distinguishing between hard and easy instances is substantially more complex due to linguistic ambiguity, sparse entity distributions, and evolving terminologies. Current mainstream research in cybersecurity NER often emphasizes downstream architectural modifications, such as adding BiLSTM and CRF layers to transformer-based models. Yet, given that models like BERT inherently encode strong contextual representations through self-attention mechanisms, such modifications have yielded limited gains. More importantly, little attention has been given to the impact of dataset composition, specifically the ratio of hard to easy data, which could directly shape the effectiveness of model fine-tuning.

To further address the challenges posed by limited annotated data, data augmentation (DA) techniques have gained increasing attention. Li et al. [19] categorize DA into paraphrasing, noising, and sampling strategies. These methods aim to improve data diversity and robustness, thereby enhancing model generalization. While federated learning and its variants [20] have been widely investigated in general domains as effective approaches to preserving both data and model privacy, their direct application in cybersecurity remains constrained. This is largely due to the difficulty of establishing alliances in which stakeholders are willing to contribute sensitive security-related data for joint model training. Synonym substitution using WordNet [21] is employed to enrich the non-entity portions of cybersecurity texts, falling within the paraphrasing category of augmentation. WordNet, a large lexical database of English, provides semantic relations such as synonyms, enabling the generation of diverse yet semantically consistent expressions. This strategy is particularly beneficial for addressing the sparsity of entities in cybersecurity datasets, which are often underrepresented but critical for robust NER performance.

In summary, this study focuses on compact and privately deployable models for cybersecurity applications. By systematically processing datasets, the proposed approach aims to optimize NER performance without introducing additional annotated data. In line with research practices observed in other domains [22], this work employs a hybrid methodology that combines PLMs with rule-based techniques to leverage the strengths of both approaches. Firstly, a hybrid discriminator is developed that integrates rule-based and deep learning methods to distinguish between hard and easy instances within cybersecurity texts. Secondly, based on the optimal hard-to-easy data ratio identified through evaluation on a curated dataset, the training data are partitioned accordingly, and data augmentation techniques are applied to the partitioned subsets to enhance the model’s ability to capture sparse entities. Finally, the augmented dataset is used as the training set to fine-tune cybersecurity NER models. The proposed method demonstrates that the ratio of hard to easy data in the training set has a significant impact on model performance and identifies an optimal balance. Furthermore, it achieves consistent improvements in the performance of cybersecurity NER, while relying on relatively small-scale datasets and lightweight models. These characteristics suggest that the proposed approach is well suited for practical deployment in sensor networks and other resource-constrained environments, where both computational efficiency and data privacy are critical.

The main contributions of this study are summarized as follows:

(1) We propose a novel approach tailored for cybersecurity Named Entity Recognition (NER) datasets, which effectively improves the accuracy of cybersecurity NER.
(2) We design a hybrid discriminator that combines DL-based and rule-based methods to identify hard and easy samples within training data. Together with a data augmentation strategy, this improves the overall quality and diversity of the training set.
(3) Our experiments demonstrate that the optimal hard to easy data ratio for cybersecurity NER is 1:1. And the proposed method not only validates this balance but also significantly enhances the ability of compact models to capture sparse entities. Moreover, the experiments are conducted on relatively small-scale datasets with lightweight models, indicating that the proposed approach is well suited for deployment in sensor networks and other resource-constrained environments.

The remainder of this paper is organized as follows. Section 2 describes the overall cybersecurity NER pipeline, including the hybrid hard–easy discriminator (rule-based and DL-based components) and the WordNet-based data augmentation strategy. Section 3 presents the experimental settings and results, focusing on the impact of different hard–easy ratios and the effect of data augmentation on sparse entities. Section 4 provides a detailed discussion of the findings in terms of dataset complexity, model architecture, and augmentation. Section 5 concludes the paper and outlines potential directions for future work.

## 2. Materials and Methods

**Figure 1:**
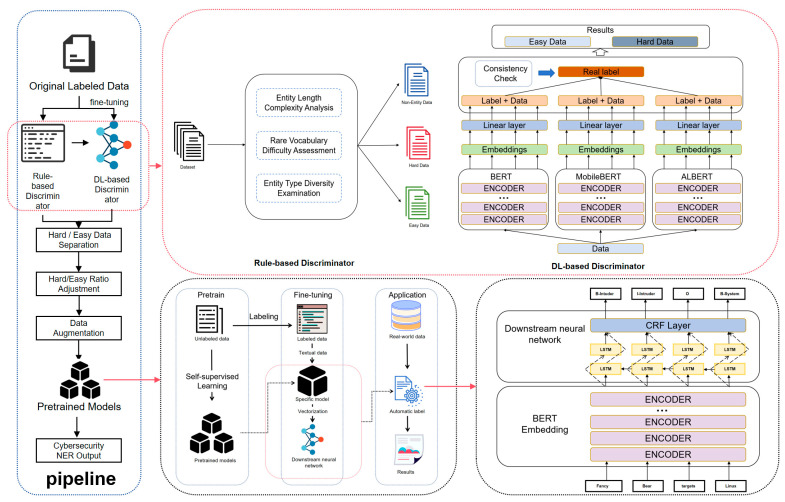
Overall Architecture.

The overall pipeline of cybersecurity NER in this study is illustrated in Figure 1, which consists of three key stages: data difficulty discrimination, ratio adjustment between hard and easy instances, and model training with augmented datasets.

In the Pipeline section, to address the challenge of distinguishing data instances with varying difficulty levels in cybersecurity texts, a hybrid discriminator composed of a Rule-based discriminator and a DL-based discriminator is incorporated as the core module. This component enables the separation of hard and easy data, allowing the training dataset to be systematically adjusted according to different hard to easy ratios. To further enhance the representation of sparse entities, a lightweight data augmentation strategy is subsequently applied to the refined dataset. This pipeline not only mitigates the adverse effects of imbalanced or ambiguous samples but also ensures improved NER performance across models of different scales under practical computational constraints.

The upper section of Figure 1 (red box) illustrates the proposed difficulty discriminator, which integrates a Rule-based Classifier and a Neural Classifier. The rule-based component evaluates sentence complexity from entity length, rarity, and type diversity, while the neural component leverages pre-trained models (BERT, ALBERT, MobileBERT) to refine the identification of hard instances. This combined strategy enables more accurate separation of hard and easy data for cybersecurity NER.

The lower section of Figure 1 (black box) illustrates the strategy for executing cybersecurity NER. The pre-training–fine-tuning paradigm is adopted to adapt general-domain models to the specific downstream task, while the combination of pre-trained models (e.g., BERT) with downstream neural layers (e.g., CRF) is employed to accomplish the final NER labeling.

The data this study utilized is annotated in the BIO format. The BIO tagging scheme is a common method used for representing named entities in sequences. In this scheme:

“B” stands for the beginning of a named entity.
“I” indicates the inside of a named entity.
“O” denotes that a token is outside any named entity.

For example, for the phrase “DroidJack RAT”, “DroidJack “would be labeled as “B-Malware”, “RAT” as “I-Malware”. This scheme helps models differentiate between entities that are adjacent in a text.

### 2.1. Rule-Based Discriminator

**Figure 2:**
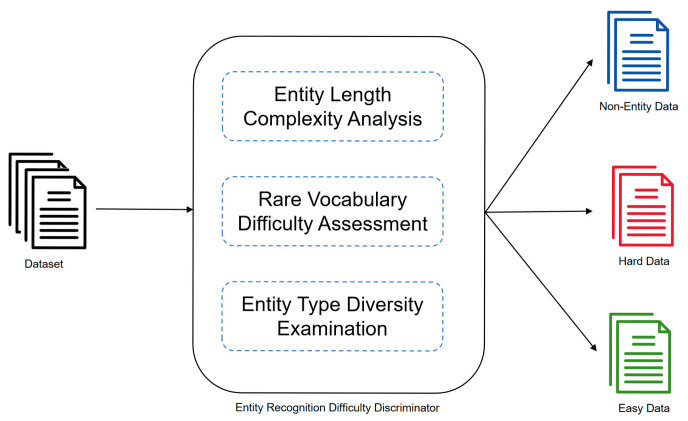
Rule-based discriminator.

The rule-based hard and easy data discriminator is illustrated in Figure 2. This component provides an initial, interpretable assessment of sentence-level difficulty in a fully rule-based and model-agnostic manner. To this end, the complexity of entities within a sentence is evaluated using three complementary difficulty metrics: entity length difficulty, rarity difficulty, and entity type diversity difficulty. These metrics exploit transparent linguistic and statistical cues to identify sentences that are potentially difficult for NER models.

Although the proposed rule-based metrics are simple, they are designed as transparent proxies for linguistic difficulty that has been repeatedly linked to human processing load and model uncertainty. Sentence- and phrase-level complexity is often operationalized through surface cues such as length and lexical difficulty in classic readability research, where average sentence length and word complexity are predictive of comprehension difficulty [23]. From a probabilistic processing view, unexpected and low-probability items contribute disproportionately to difficulty, which motivates measuring lexical rarity as a proxy for predictive uncertainty [24]. Finally, in NER—especially in domain text like cyber threat intelligence—errors frequently arise from boundary ambiguity and label/type confusion; therefore, we explicitly operationalize span-length and type-mixing signals as interpretable difficulty indicators [25].

1. Entity Length Difficulty: This metric measures aggregate additional length beyond single-token entities. Longer mentions enlarge the boundary search space and increase boundary ambiguity, which is a recognized difficulty factor in span-based and boundary-aware NER models. This is particularly relevant when entities are multi-token or structurally complex, where boundary errors become more frequent and dedicated boundary-enhancement methods have been proposed [25]. The calculation involves summing the total number of words across all entities and subtracting the number of entities, thereby producing a value indicative of entity length complexity. This approach emphasizes the additional difficulty posed by multi-word entities beyond merely counting the total number of entities. Formally, let a sentence contain N entities, and let $L_{i}$ denote the number of words in the i-th entity. The entity length difficulty is defined as:

(1) $$D_{length}=\left(\sum_{i=1}^{N}L_{i}\right)-N$$

This formulation emphasizes the additional difficulty posed by multi-word entities beyond merely counting the total number of entities.

2. Rarity Difficulty: This metric captures the influence of uncommon vocabulary within entities. Entities containing rare terms are more challenging to identify accurately, often due to insufficient contextual evidence or limited model familiarity with such terms. Word frequency is examined within each entity, and terms whose frequency falls below an automatically determined threshold are treated as rare, contributing to the rarity score. Lexical rarity is a well-established source of processing difficulty: low-frequency words are generally harder to recognize and process than high-frequency words [26]. In NER, rare entity surface forms are also a known failure mode; recent work explicitly targets out-of-vocabulary entity recognition [27]. This issue is amplified in cybersecurity text, where many entity strings (e.g., malwares, tools, campaign names) are inherently sparse and “rare-entity recognition” is called out as a key challenge in prior cybersecurity NER research [28].

Instead of manually specifying a rarity threshold, we determine the threshold τ automatically from the data using the mean frequency. Let S denote the set of sentences that contain at least one entity, and let $W=\{w_{1},w_{2},\ldots ,w_{M}\}$ be the multiset of all words that appear inside entities across all sentences in S. Each word w∈W is associated with a corpus-level frequency f(w). The rarity threshold τ is then defined as the average frequency of these words:

(2) $$\tau =\frac{1}{\left|W\right|}\sum_{w\in W}f(w)$$

Given a specific sentence, suppose the set of words contained in all entities of that sentence is $\left\{w_{1},w_{2},\ldots ,w_{M}\right\}$ with corresponding frequencies $f(w_{j})$ for each word $w_{j}$, Words whose frequency falls below the automatically determined threshold *τ* are considered rare. The rarity difficulty for this sentence is then defined as:

(3) $$D_{rarity}=\sum_{j=1}^{M}I(f(w_{j})<\tau )$$

where I() is the indicator function that equals 1 if the condition is satisfied and 0 otherwise.

3. Entity Type Diversity Difficulty: This metric evaluates the variety of entity categories present within a sentence, as denoted by the “B-” or “I-” prefixes in labels, A larger number of distinct entity types suggests higher structural and semantic complexity, which increases recognition difficulty. In this framework, a set of entity types is constructed for each sentence, and the cardinality of this set quantifies the entity type diversity difficulty. A higher number of distinct entity types within the same sentence increases type competition and label confusion, which aligns with error analyses showing that label mismatch is a major category of NER errors alongside span mismatch [29]. Let T denote the set of distinct entity types observed in the sentence (e.g., B-Malware, I-Malware) The entity type diversity difficulty is defined as:

(4) $$D_{diversity}=|T|$$

where |T| denotes the cardinality of the entity type set.

By summing the values of these three metrics, a total difficulty score is obtained to gauge the overall complexity of a sentence. Comparison of this composite score with a data-driven threshold enables the identification of sentences more likely to contain entities that are difficult to recognize, thereby classifying them as hard data. the overall difficulty score is obtained by summing the three individual components:

(5) $$D_{total}=D_{length}+D_{rarity}+D_{diversity}$$

The threshold is determined automatically from the dataset. Specifically, let S denote the set of sentences that contain at least one entity, and let |S| be its cardinality. The adaptive threshold is defined as the average total difficulty score across all such sentences:

(6) $$\theta =\frac{1}{\left|S\right|}\sum_{s\in S}D_{total}(s)$$

where $D_{total}(s)$ denotes the total difficulty score for sentence s.

The classification rule is then given by:

(7) $$\begin{array}{c} Classification=\left\{\begin{array}{ll} Hard, & D_{total}\geq \theta \\ Easy, & D_{total}<\theta \end{array}\right. \end{array}$$

From a computational perspective, the proposed rule-based discriminator is lightweight. For a sentence of length L containing N entities, the computation of the three difficulty metrics $D_{length}$, $D_{rarity}$, $D_{diversity}$ as well as the total difficulty score $D_{total}$ and the subsequent hard/easy classification, requires only a single pass over the tokens. Each step involves constant-time operations such as counting token positions, looking up pre-computed word frequencies, and updating a small set of entity types, resulting in an overall time complexity of O(L) per sentence. Corpus-level estimation of τ and θ adds one linear pass over the dataset, i.e., O(T) where $T=O(\sum_{s\in S}L_{s})$, $L_{s}$ is the total number of tokens. Since this rule-based module is executed once as a preprocessing step before model training and does not participate in inference, its overhead is negligible compared with the repeated forward–backward passes of Transformer-based NER models in subsequent training epochs.

We acknowledge that threshold-based heuristics can be corpus-dependent, because lexical frequency distributions and entity compositional patterns may differ across domains and datasets. The proposed three difficulty metrics are intended as interpretable proxies of NER difficulty rather than an exhaustive characterization of linguistic complexity: (i) longer multi-token entities typically increase span-boundary ambiguity; (ii) rare entity tokens reflect higher lexical uncertainty and reduced exposure, which is particularly common in cybersecurity text with emerging jargon and novel names; and (iii) higher entity-type diversity increases labeling heterogeneity and the risk of type confusion within a sentence. To reduce reliance on manually chosen cutoffs and improve applicability across corpora, we adopt data-driven thresholds computed from corpus statistics (Equations (2) and (6)), which define “hard” examples relative to the target corpus. We also note that further improving cross-corpus transferability (e.g., via robust quantile-based thresholding or corpus-adaptive calibration) is a promising direction for future work.

The contribution of this rule-based component is to provide an interpretable, model-independent mechanism that exploits linguistic length, lexical rarity, and label diversity signals to produce an initial hard/easy partition of the data at the sentence level.

### 2.2. DL-Based Discriminator

**Figure 3:**
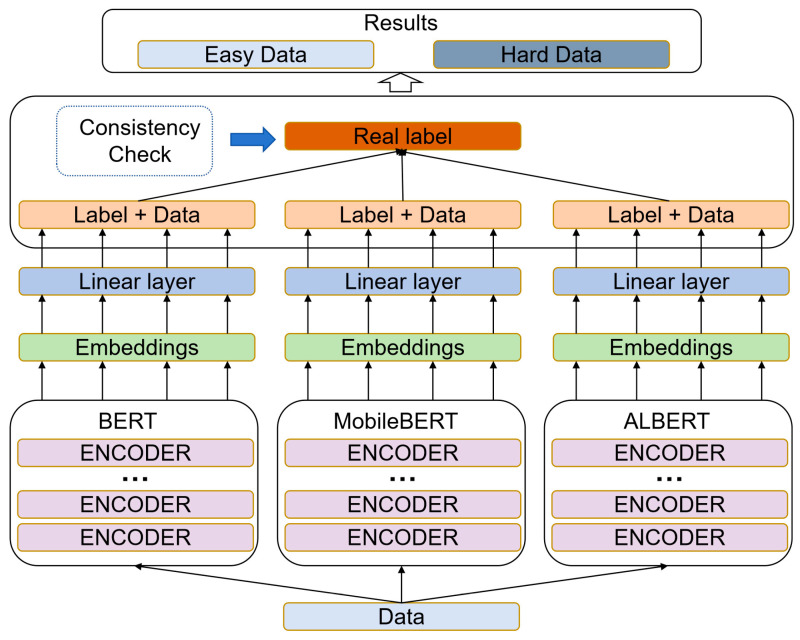
DL-based discriminator.

The DL-based data discriminator is illustrated in Figure 3. This component refines the initial rule-based labels using a set of heterogeneous NER models, thereby introducing a data-driven, ensemble-based validation step that mitigates model-specific biases and reduces false positives in the hard set.

Following the preliminary rule-based assessment, three named entity recognition models of varying scales and architectures—BERT, ALBERT, and MobileBERT—are employed to further filter the hard data identified in the preceding step.

BERT is a bidirectional Transformer model pre-trained on large-scale corpora using masked language modeling and next sentence prediction. It achieves strong performance across a wide range of NLP tasks, but its large parameter size makes it computationally demanding. MobileBERT is a compact variant of BERT optimized for mobile and resource-limited environments. Through knowledge distillation and architectural modifications such as bottleneck layers, it reduces latency and model size while maintaining performance comparable to BERT. ALBERT is designed to improve efficiency by reducing parameters and training time. It introduces factorized embedding parameterization and cross-layer parameter sharing, resulting in a lightweight model that retains competitive accuracy despite its smaller size.

After extracting contextual embeddings from BERT, ALBERT, and MobileBERT, a linear classification layer was applied for the NER task. Given a token sequence $\left(x_{1},x_{2},\ldots ,x_{T}\right)$ each model produces contextual representations

(8) $$h_{t}\in R^{d},\;t=1,\ldots ,T,$$

where d denotes the hidden dimension of the model.

The linear classifier projects these embeddings into predefined entity categories via a softmax layer:

(9) $$y_{t}=softmax(Wh_{t}+b)$$

where W and b are trainable parameters, and $y_{t}$ represents the predicted probability distribution over NER labels for token $x_{t}$. Each model was fine-tuned on a labeled NER dataset to align the contextual embeddings with entity classification objectives. Following fine-tuning, the three models were used to validate the “hard” data identified by the rule-based discriminator. Entity recognition was performed independently by each model, and the resulting label assignments were compared. Let ${\hat{y}}_{t}^{m}$ denote the label predicted for token $x_{t}$ by model m∈{1,2,3}. An instance was conclusively classified as hard data if at least two models agreed on an incorrect label assignment:

(10) $$\exists \;t\;\;s.t.\sum_{m=1}^{3}I({\hat{y}}_{t}^{m}\neq y_{t})\geq 2$$

where $y_{t}$ denotes the true label and I() is the indicator function. Conversely, if all three models produced correct predictions across the entity span, the instance was reclassified as easy data.

From a computational standpoint, the DL-based discriminator introduces only moderate training-time overhead. For a sentence of length T, each backbone model (BERT, ALBERT, MobileBERT) performs a standard forward pass with time complexity comparable to that of conventional Transformer-based NER, and the ensemble step simply repeats this operation three times on the same input. Let |H| denote the number of sentences initially flagged as hard by the rule-based discriminator; the total cost of DL-based validation is thus proportional to 3 ∗ |H|. This validation is executed once as an additional training-time procedure and is not invoked during inference. At test time, the online decision complexity remains identical to that of the chosen NER backbone, so the DL-based discriminator does not affect runtime behavior in deployment scenarios.

The contribution of this DL-based component is to introduce a data-driven, ensemble-based validation mechanism that leverages the complementary strengths of heterogeneous NER architectures (BERT, ALBERT, MobileBERT). It helps filter out false positives generated by the rule-based discriminator and focuses the hard set on instances that are empirically difficult for modern NER models, thus improving the reliability and robustness of the hard–easy discrimination.

In summary, the rule-based discriminator provides an interpretable, model-agnostic initial screening of sentence difficulty, while the DL-based discriminator performs a data-driven, ensemble validation and refinement of these labels. Together, these two components yield a more reliable and robust hard–easy discrimination than either component alone.

### 2.3. Data Augmentation

After applying the two discriminators and adjusting the ratio of hard to easy samples in the training set, the overall dataset size was inevitably reduced. This reduction arises because certain sentences were excluded as non-informative or reclassified in order to achieve the intended distributional balance between hard and easy data. While such filtering improves dataset quality, it also leads to a smaller training corpus, which may weaken the model’s capacity to generalize, particularly in covering rare entities or long-tail contexts.

To address this issue, data augmentation was introduced. The key objective was to restore the total dataset size to the same level as before filtering, thereby maintaining the statistical comparability of experiments while also enriching the diversity of sentence formulations. In this way, augmentation compensates for the loss of volume caused by prior selection and balancing steps, ensuring that the training procedure benefits both from improved difficulty calibration and from sufficient data quantity.

Concretely, data augmentation is performed at the token level on the BIO-labeled training corpus. In this study, we adopted a WordNet-based augmentation strategy. WordNet is a large lexical database of English that organizes words into sets of cognitive synonyms and encodes various semantic relations among them (e.g., synonymy and hypernymy), which allows us to obtain semantically related replacement candidates for ordinary words. For each sentence, we keep the original BIO labels unchanged. All tokens tagged with “B-” or “I-” are left intact so that the surface forms and boundaries of cybersecurity entities remain unchanged. For each token labeled as “O”, we query WordNet to obtain its synsets and extract candidate synonyms; when at least one suitable synonym is found, the original token is replaced by one of these synonyms, while the “O” label is preserved. This procedure generates one augmented counterpart for each original sentence in the training set. Using this method, the original dataset with 71,002 BIO-labeled token–label pairs is expanded to a total of 142,002 pairs.

**Figure 4:**
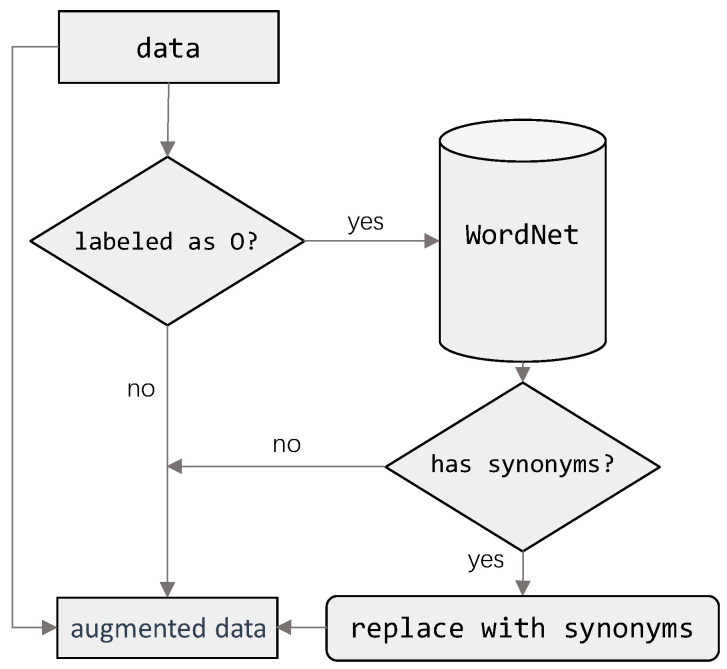
Data Augmentation Architecture.

The data augmentation framework is illustrated in Figure 4. This design is particularly important for cybersecurity text, where indicators frequently appear as rigid identifiers such as MD5 hash strings (32-character hexadecimal representations of MD5 cryptographic hashes used to identify files) or other highly specialized tokens. Substituting synonyms for such entities may produce semantically invalid or misleading results (e.g., replacing the system term Android with Humanoid robot). By only modifying non-entity tokens and preserving all “B-” and “I-” tokens, the augmentation process diversifies the surrounding context while keeping entity semantics intact. Model performance under this augmentation regime is subsequently evaluated using standard metrics, namely precision, recall, and F-measure, computed from true positives (*TP*), true negatives (*TN*), false positives (*FP*), and false negatives (*FN*).

From a computational perspective, the proposed augmentation procedure is lightweight. Since augmentation is performed once as an offline preprocessing step on the BIO-labeled training corpus, its time complexity is linear in the number of tokens: for each sentence, the algorithm scans the sequence a single time, checks the BIO label of each token, and, only for tokens labeled “O”, performs a limited number of WordNet lookups and synonym substitutions. Let T denote the total number of tokens in the training set; the overall cost of augmentation is therefore O(T), which is negligible compared with the repeated forward–backward passes of the downstream NER models during training.

## 3. Results

### 3.1. Experimental Settings

**Figure 5:**
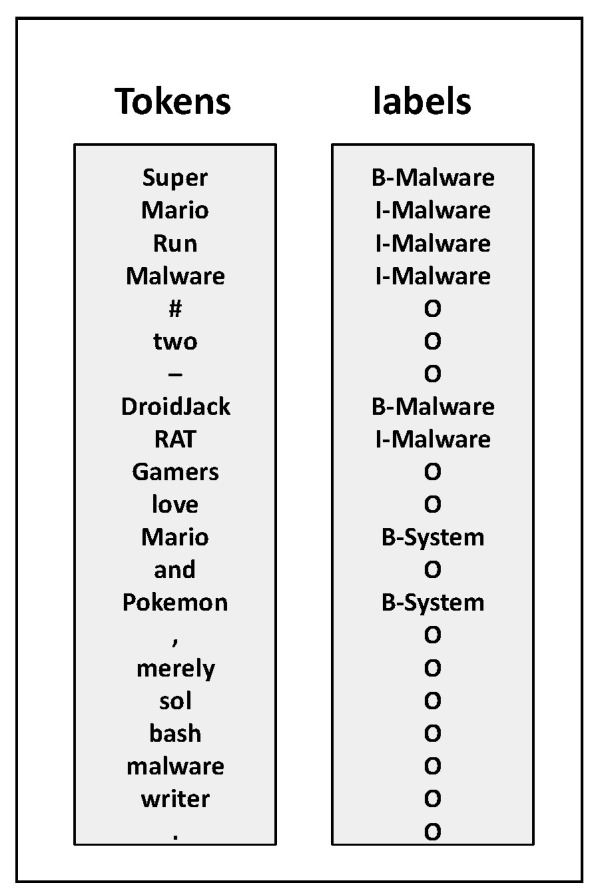
Data Sample.

The dataset employed in this study is a publicly available corpus introduced by M. T. Alam and colleagues in CyNER: A Python Library for Cybersecurity Named Entity Recognition [11]. It was constructed from approximately 60 threat intelligence reports obtained from the MITRE ATT&CK website under the software category, with each report corresponding to a distinct malware instance. The original authors manually extracted and cleaned the text from these reports and subsequently annotated the corpus using the BRAT annotation tool developed by Stenetorp et al. [30]. Annotation follows the BIO tagging scheme, as illustrated in Figure 5. The dataset encompasses five categories of cybersecurity entities—Indicator, Malware, Organization, System, and Vulnerability. Owing to the BIO scheme, this results in a total of 11 unique labels. The distribution of entity types across the corpus is summarized in Table 1.

The parameters of the two different lightweight pre-trained models used in the experiment are shown in Table 2. For comparison, the table also provides the size and number of parameters of the original BERT model.

Similar to CyNER, each model is trained for a total of 20 epochs, utilizing a sequence length set at 128. For the pre-trained models, we adopt an initial learning rate of 10^−5^. Training batches consist of 32 samples. The AdamW optimizer, as proposed by Loshchilov and Hutter [31], is employed throughout the training process, and all computations are executed on a singular Nvidia RTX 4090 GPU. We conducted two sets of experiments on each lightweight pre-trained model using both the data before and after augmentation.

### 3.2. Impact of Hard and Easy Data on NER Performance in Training Sets

Consistent with common practice, the dataset was divided into training, validation, and test sets with an 8:1:1 ratio. The training data were used to fine-tune pre-trained language models, in combination with a rule-based discriminator and a DL-based discriminator, to classify data into hard and easy subsets. In the first step, entities with above-average complexity were provisionally designated as hard data by the rule-based discriminator. These instances were subsequently evaluated by the DL-based discriminator, and if two or more models misclassified an entity, it was conclusively labeled as hard data; the remaining entities were classified as easy data. This procedure first yielded two entity-level subsets, a hard entity set and an easy entity set. Since the NER model operates at the sentence level, we then constructed sentence-level training data from these subsets: sentences containing at least one hard (respectively, easy) entity were assigned to the hard (respectively, easy) training set. After applying this discrimination and construction procedure, the easy subset comprises 59,033 token–label pairs and the hard subset comprises 41,656 pairs. Note that the two subsets are overlapping, because sentences that contain both hard and easy entities contribute token–label pairs to both subsets. The intersection includes 29,687 pairs (≈41.8% of all tokens).

**Figure 6:**
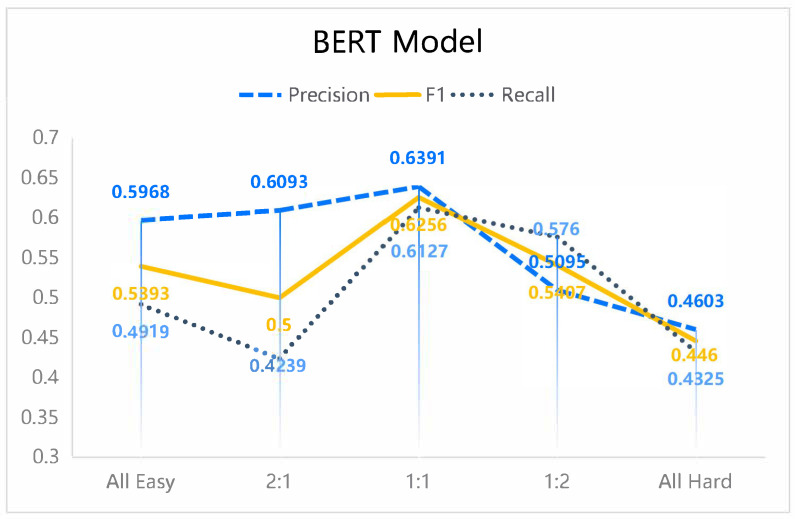
The NER performance of the BERT model under varying ratios of hard to easy data in the training dataset.

**Figure 7:**
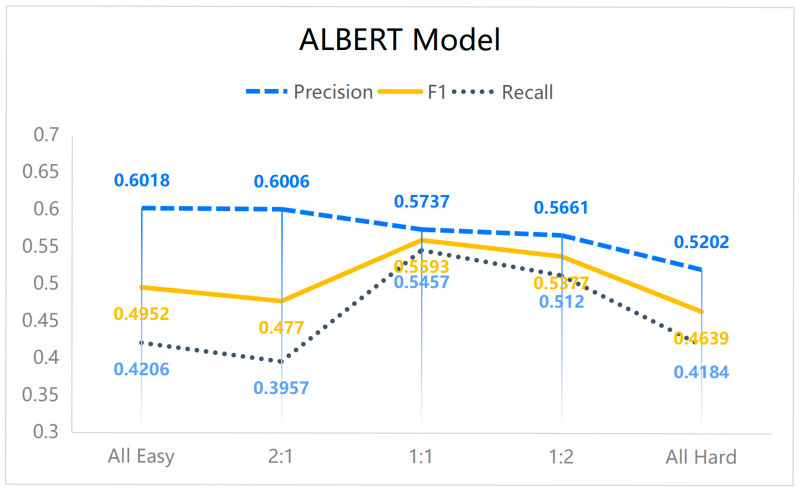
The NER performance of the ALBERT model under varying ratios of hard to easy data in the training dataset.

**Figure 8:**
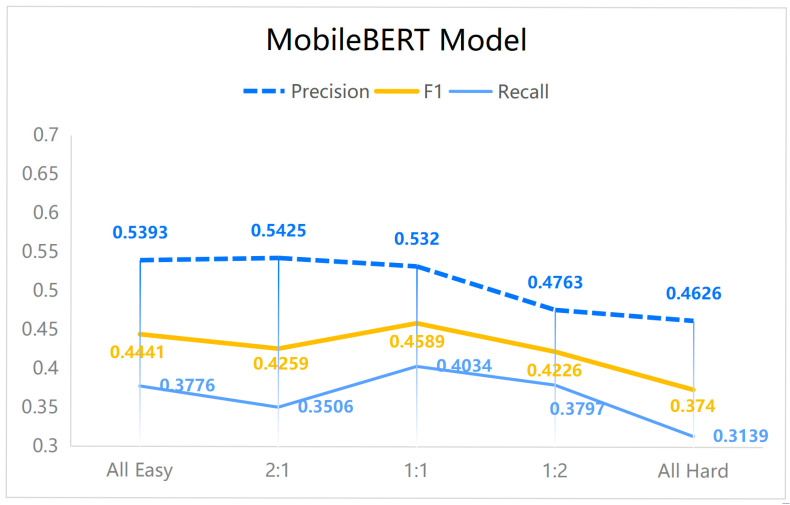
The NER performance of the MobileBERT model under varying ratios of hard to easy data in the training dataset.

Based on these subsets, five different configurations were constructed with varying proportions of hard to easy data (all-easy, all-hard, 1:2, 1:1, and 2:1), while maintaining a nearly constant overall number of entities. These configurations were used to retrain three models, with results presented in Figure 6, Figure 7 and Figure 8. Importantly, the validation and test sets remained unchanged, containing both hard and easy data to preserve realistic evaluation conditions. Since the dataset consists of 60 authentic cybersecurity reports, the outcomes provide insight into model performance under real-world scenarios, demonstrating the applicability and reliability of the proposed approach.

During experimentation, it was observed that smaller models (ALBERT and MobileBERT) exhibited weaker feature extraction capabilities for sparse entity categories, such as Vulnerability, compared to the larger BERT model. To mitigate this limitation, a WordNet-based data augmentation method was applied to enrich the training set, substantially improving the performance of smaller models on sparse entities.

Model performance was evaluated using precision, recall, and F1-score, defined as follows:

(11) $$\begin{array}{c} Precision=\frac{TP}{TP+FP},Recall=\frac{TP}{TP+FN},F1=\frac{2\cdot Precision\cdot Recall}{Precision+Recall} \end{array}$$

In this section, our main focus is to investigate how the proportion of hard and easy data in the training set affects model performance. The datasets used in this part are subsets of the original dataset defined above; therefore, the resulting performance is inevitably lower than that obtained when training on the full original dataset (Table 3). The experimental results (with random seed 1) demonstrate that, for the task of cybersecurity named entity recognition, all three models achieved superior F1-scores, recall, and precision when trained on data with a 1:1 ratio of hard to easy instances. Although this configuration did not consistently yield the highest accuracy, the substantial gains in F1 and recall are particularly significant for cybersecurity applications. These metrics are critical in this domain, as they not only capture the ability of a model to correctly identify true entities (precision) but also its effectiveness in minimizing missed detections (recall), thereby supporting balanced performance essential for reliable threat identification.

The findings further reveal that training exclusively on easy data can lead to inflated accuracy but reduced F1 and recall. This effect arises because the model fails to adequately capture complex entities, particularly when sentences contain multiple distinct entities or when a single entity spans multiple labels. It is noteworthy that in the experimental design, each training set—across the five ratios of hard to easy data—contained nearly equal numbers of entities. Consequently, an increase in hard data necessarily entailed a reduction in easy data, preserving a consistent total entity count across conditions. This balance enabled a controlled evaluation of model adaptability and performance under varying levels of data complexity.

To reflect the variability and reliability of the results, all experiments in this section were repeated with multiple random seeds, and we report averages together with 95% confidence intervals. For the MobileBERT model, five runs with seeds {2, 10, 50, 100, 1000} yielded F1-scores of 0.47, 0.42, 0.45, 0.40, and 0.46, respectively, resulting in an average F1 of 0.44 with a 95% confidence interval of [0.40, 0.48]; the corresponding recall is 0.38 [0.35, 0.41] and precision is 0.52 [0.49, 0.56]. Similar behavior was observed for the other backbone models and hard–easy ratios, where the widths of the 95% confidence intervals were generally below 0.06. These relatively narrow intervals indicate that the proposed difficulty-aware training strategy yields stable performance across random initializations.

Overall, the results underscore the importance of dataset complexity as a factor distinct from dataset size. The influence of complexity on model performance is non-trivial and should be carefully considered in future studies on named entity recognition and related tasks.

### 3.3. Ablation Study: Effect of Data Augmentation on Sparse Entity Types

The experiments also revealed a challenge unique to cybersecurity named entities: certain categories, such as Vulnerability, are highly distinctive yet occur with extreme rarity. In the publicly available dataset employed in this study, the distribution of entities in the training set closely mirrors that of the test set. Specifically, the counts for Malware, Indicator, System, and Organization are 703, 1021, 837, and 284, respectively, whereas Vulnerability appears only 48 times. Given that the corpus is derived from MITRE’s ATT&CK cybersecurity reports, this imbalance is likely reflective of an inherent characteristic of real-world cybersecurity data. To isolate the contribution of the augmentation module, we perform an ablation experiment by removing data augmentation while keeping the backbone NER model, training hyperparameters, hard–easy selection strategy, and discriminators unchanged.

Model performance exhibited substantial variation across entity types depending on model size. Large-scale architectures such as BERT were able to capture features of rare entities relatively well, whereas smaller models such as ALBERT and MobileBERT performed poorly, highlighting the critical role of entity rarity in model selection for cybersecurity NER tasks. To mitigate this issue and enhance the utility of smaller pre-trained models, we applied the simple synonym-based data augmentation strategy described in Section 2.3, which increases the frequency of rare entities in the training set. Using this method, the original dataset with 71,002 BIO-labeled token–label pairs was augmented to a total of 142,002 pairs.

**Figure 9:**
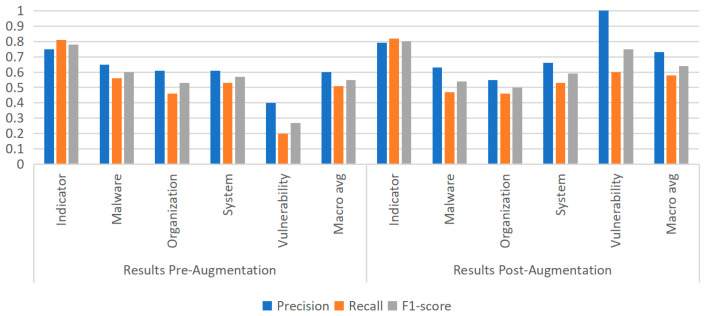
Experimental results of the ALBERT model before and after data augmentation.

**Figure 10:**
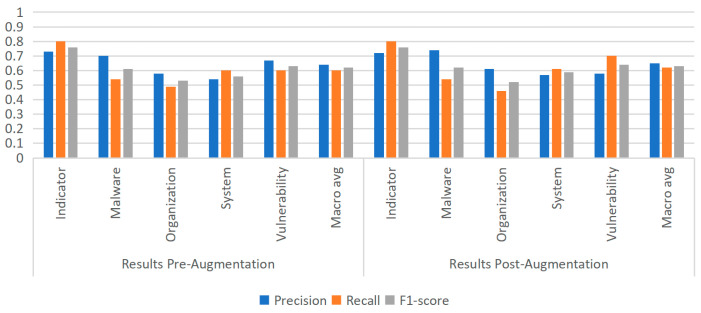
Experimental results of the MobileBERT model before and after data augmentation.

**Figure 11:**
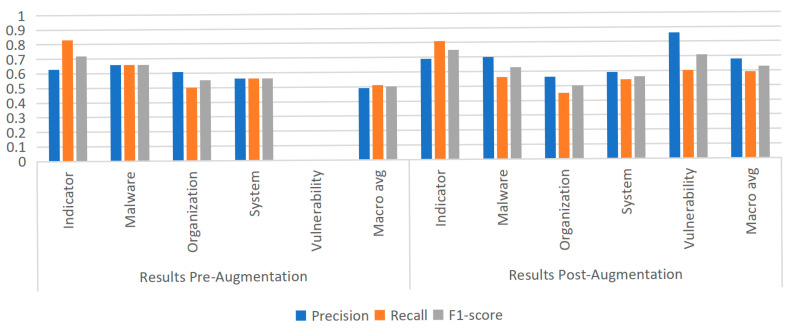
Experimental results of the MiniLM model before and after data augmentation.

To further examine the generality of this phenomenon, additional experiments were conducted using the MiniLM model, thereby extending the investigation to a broader range of architectures. Following augmentation, experiments were carried out to evaluate its impact on model performance. As shown in Figure 9, Figure 10 and Figure 11, under identical training and evaluation settings, removing data augmentation consistently degrades performance, particularly on the rare Vulnerability category; reintroducing augmentation restores and further improves performance across multiple entity types. These findings confirm the effectiveness of the proposed strategy and provide broader insights into addressing both entity rarity and overall robustness in cybersecurity NER. In addition, for reference, we report the results of existing approaches on the same dataset in Table 4 under the same evaluation protocol; together with our experiments, these comparative results confirm the effectiveness of the proposed strategy and provide broader insights into addressing both entity rarity and overall robustness in cybersecurity NER.

Analysis of the experimental results indicates that compact pre-trained models, derived through different methodologies, exhibit varying levels of effectiveness in cybersecurity named entity recognition. The MiniLM models, distilled from the original BERT, achieve performance broadly consistent with their teacher model. MobileBERT, optimized for deployment on mobile devices through a distinctive bottleneck architecture and uniform scaling rules, demonstrates superior performance relative to MiniLM. However, it was not explicitly designed to address limited-data scenarios. In contrast, ALBERT introduces cross-layer parameter sharing and replaces BERT’s Next Sentence Prediction (NSP) objective with a Sentence Order Prediction (SOP) task. This architectural departure makes ALBERT particularly suitable for smaller datasets, a trend reflected in its superior empirical performance.

Despite these differences, all three compact models—MobileBERT, ALBERT, and MiniLM—share a common limitation: reduced capacity for capturing sparse entity types. The experiments, however, show that this weakness can be substantially mitigated through the combined use of the proposed hard–easy data selection strategy and a straightforward data augmentation method. These findings underscore the value and practicality of pursuing compact models for cybersecurity NER, given their efficiency and applicability in real-world settings. Moreover, they highlight the importance of continued research into augmentation techniques and their potential to further improve model robustness and performance.

## 4. Discussion

The findings of this study provide several insights into the role of dataset complexity, model architecture, and data augmentation in cybersecurity named entity recognition (NER).

First, the results demonstrate that dataset composition, specifically the balance between hard and easy instances, substantially influences model performance. A 1:1 ratio of hard to easy data yielded the best trade-off between precision and recall, leading to the highest F1-scores across models. While training exclusively on easy data produced slightly higher accuracy, this came at the cost of reduced recall and F1, underscoring the importance of exposing models to sufficiently complex instances. These observations highlight dataset complexity as a critical factor that must be considered alongside dataset size in the design of cybersecurity NER systems.

Second, the comparative analysis of compact pre-trained models revealed distinct performance characteristics linked to their architectural design. MiniLM, derived through knowledge distillation from BERT, displayed performance broadly aligned with its teacher model. MobileBERT, optimized for deployment on resource-constrained devices through a bottleneck architecture and scaling rules, outperformed MiniLM in most settings. However, it was not specifically designed to handle limited-data scenarios. ALBERT, which introduces cross-layer parameter sharing and replaces the NSP objective with SOP, proved particularly effective for smaller datasets and achieved the strongest overall performance in our experiments. These differences illustrate the influence of architectural choices on model adaptability to varying levels of data complexity.

Third, a notable challenge in cybersecurity NER lies in the highly imbalanced distribution of entity categories. Rare but distinctive types such as “Vulnerability” occur with very low frequency in comparison to more common entities such as “Malware” or “Indicator”. Large-scale models were able to capture features of these rare entities, whereas smaller models exhibited significant limitations. This weakness, however, can be substantially mitigated through the combined use of the proposed hard–easy data selection strategy and a straightforward data augmentation method. By increasing the representation of sparse entities while maintaining a balanced overall dataset size, the augmentation process improved performance not only on rare categories but also on other entity types, enhancing the robustness of all models evaluated.

Fourth, we briefly discuss the sensitivity of the proposed method with respect to different conditions and assumptions. Varying the hard–easy ratio (see Figure 6, Figure 7 and Figure 8) revealed that our approach is robust as long as both subsets remain sufficiently represented: extreme settings such as “all-easy” or “all-hard” consistently underperformed, whereas intermediate ratios around 1:1 yielded stable and superior results across models. In addition, although the absolute performance levels differ among backbone architectures (MiniLM, MobileBERT, ALBERT), the relative gains obtained by introducing difficulty-aware data selection and augmentation were observed for all of them, suggesting that the method is not tied to a specific model design but rather acts as a generic training-time enhancement. Finally, because the rarity and difficulty thresholds are computed in a data-driven manner, the framework adapts to different corpus statistics (e.g., varying entity distributions and sentence lengths), which reduces its sensitivity to manual hyperparameter choices while still allowing the hard–easy split to reflect dataset-specific characteristics.

Finally, we acknowledge several limitations of the proposed framework, which point to promising avenues for future work. First, our experiments are conducted on a single English cybersecurity corpus, so the generalizability of the hard–easy discrimination strategy to other domains and languages remains to be fully validated; future studies will extend the evaluation to additional security-related and multilingual NER datasets and adapt the difficulty metrics to alternative annotation schemes. Second, the rule-based discriminator is built on three hand-crafted, sentence-level indicators (length, rarity, and entity-type diversity), which, while interpretable, may not fully capture more subtle forms of semantic ambiguity or contextual difficulty. A natural extension is to combine these metrics with learned difficulty signals derived from model uncertainty or meta-features. Third, the current WordNet-based augmentation is limited to synonym substitution for non-entity tokens and to English text; exploring more advanced, context-aware augmentation methods (e.g., back-translation, masked language model rewriting, or generative paraphrasing) and multilingual resources could further enhance robustness. Finally, our DL-based discriminator is implemented using multiple backbone models, which inevitably adds training-time overhead. As future work, we plan to improve efficiency by distilling the ensemble into a single compact difficulty predictor, or by learning difficulty signals jointly with the NER model in an end-to-end framework. We also note that the rule-based and DL-based discriminators are intended to work together as a two-stage screening component; therefore, a discriminator-only ablation that disentangles their individual effects is deferred to future work.

## 5. Conclusions

This study emphasizes the practical implications of these findings. Given that the dataset is derived from authentic cybersecurity threat intelligence reports, the experimental results reflect realistic challenges encountered in operational settings. The demonstrated effectiveness of compact models, once supplemented with principled data selection and augmentation strategies, suggests their viability for real-world deployment on sensor devices, edge nodes, and intrusion detection sensors, particularly in environments with strict resource constraints and privacy requirements. At the same time, the results underscore the need for continued research into adaptive data sampling and augmentation techniques tailored to domain-specific challenges such as entity rarity, contextual ambiguity, and label complexity.

In summary, this work establishes dataset complexity as a central dimension of cybersecurity NER research, highlights the distinct advantages and limitations of compact model architectures, and demonstrates that the combination of hard–easy data selection with augmentation can serve as a practical mechanism to address model weaknesses. Future work will extend these findings by exploring more sophisticated augmentation methods, investigating cross-domain generalization across different types of sensor deployments, and assessing the applicability of these strategies to other security-related NLP tasks that operate on text derived from sensor systems and sensor networks.

## Figures and Tables

**Table 1:** Distribution of Entity Types within the Dataset.

Entity Type	Entity Count	Percentage
Indicator	1490	32.9%
Malware	1199	26.4%
Organization	508	11.2%
System	1267	28.0%
Vulnerability	67	1.5%

**Table 2:** Model parameters and sizes.

Model Name	Model Size (MB)	Parameter Count (Million)
BERT-base-uncase	440	110
AlBERT-base-v2	47	11
MobileBERT-uncase	147	25

**Table 3:** Performance of the base models on the initial dataset.

Model Name	Precision	Recall	F1-Score
BERT-base-uncase	0.64	0.68	0.66
MobileBERT	0.64	0.6	0.62
ALBERT	0.6	0.51	0.55

**Table 4:** Performance of the other approaches on the same dataset.

Model Name	Precision	Recall	F1-Score
BERT-base-uncased	0.69	0.69	0.69
BERT-large-uncased	0.72	0.73	0.73
RoBERTa-base	0.37	0.42	0.39
RoBERTa-large	0.34	0.44	0.38

## Data Availability

The dataset used in this study is publicly accessible, and its source has been described in the main text. Additional requests may be directed to the corresponding author.
